# Frequency and associated factors of hepatic hemangioma in systemic lupus erythematosus patients undergoing abdominal imaging: a retrospective cohort study

**DOI:** 10.1007/s00296-026-06164-7

**Published:** 2026-05-28

**Authors:** Döndü Üsküdar Cansu, Burcu Ceren Uludoğan, Reşit Yıldırım, Gamze Önal Birtane, Taner Yılmaz, Ufuktan Özbayrak, Cengiz Korkmaz

**Affiliations:** 1 Division of Rheumatology, Department of Internal Medicine, Faculty of Medicine, Eskişehir Osmangazi University, 26480 Eskişehir, Turkey; 2 The Royal Wolverhampton NHS Trust, Wolverhampton, UK; 3 University of Milan, Milan, Italy

**Keywords:** Systemic lupus erythematosus, Hemangioma, Liver, Anemia, Hemolytic, Autoimmune, Pleurisy, Ultrasonography

## Abstract

Hepatic hemangioma may be detected in patients with systemic lupus erythematosus (SLE). This study aimed to determine the frequency, radiological characteristics, and potential associated factors of hepatic hemangioma in SLE patients undergoing abdominal imaging. We retrospectively evaluated 221 of 402 SLE patients followed at a tertiary university hospital who underwent abdominal imaging for clinical indications. Demographic, clinical, laboratory, and radiological data were collected from medical records. Factors associated with hepatic hemangioma were analyzed. A total of 221 SLE patients who underwent abdominal imaging were included. All patients underwent abdominal ultrasound as the primary imaging modality. The mean age was 49.62 ± 13.75 years, 86.4% were female, mean age at SLE diagnosis was 36.29 ± 14.48 years, and mean disease duration was 12.81 ± 8.14 years. The most common indication for abdominal imaging was asymptomatic/routine screening/pre-renal biopsy evaluation (n = 117, 53%). Hepatic hemangioma was detected in 11.8% (n = 26) of the imaged SLE patients. Among these, 69.2% were identified after SLE diagnosis, 27% concurrently, and 3.8% before diagnosis. Most hemangiomas were solitary (65.4%), with a mean diameter of 2.57 ± 2.22 cm. Patients with hepatic hemangioma had significantly longer disease duration (15.85 ± 7.82 vs. 12.40 ± 8.12 years; p = 0.043) and follow-up duration (15.38 ± 7.98 vs. 11.61 ± 7.89 years; p = 0.030) compared to those without. At SLE diagnosis, pleuritis (19.2% vs. 5.6%; p = 0.035), autoimmune hemolytic anemia (AIHA) (38.5% vs. 18.6%; p = 0.037), and C3 hypocomplementemia (80.8% vs. 55.4%; p = 0.014) were significantly more common in the hemangioma group. Multivariate logistic regression analysis revealed that longer SLE disease duration (OR 1.06 per year; 95% CI 1.01–1.12; p = 0.020) and presence of AIHA at diagnosis (OR 3.42; 95% CI 1.32–8.88; p = 0.011) were independently associated with hepatic hemangioma. Hepatic hemangiomas may be incidentally detected in SLE patients undergoing abdominal imaging. They were significantly more frequent in patients with longer disease duration and in those with AIHA at SLE diagnosis. However, these findings should be interpreted cautiously due to the retrospective design and selected nature of the study population. Prospective longitudinal studies with unselected SLE cohorts and appropriate control groups are needed to better define the true frequency and their associated factors of hepatic hemangiomas in SLE.

**Supplementary Information:**

The online version contains supplementary material available at 10.1007/s00296-026-06164-7.

## Introduction

Systemic lupus erythematosus (SLE) is a systemic connective tissue disease characterized by chronic inflammation and autoimmune mechanisms, involving multiple organs. Although liver involvement is relatively uncommon in SLE, it may present across a broad clinical spectrum. While autoimmune hepatitis is a well-recognized manifestation of SLE, severe hepatotoxicity related to certain therapeutic agents and acute liver failure presenting as the initial manifestation of SLE have also been described [1–3]. In contrast, data regarding structural or vascular liver lesions in SLE remain quite limited. Hepatic manifestations such as nodular regenerative hyperplasia, Budd-Chiari syndrome, focal nodular hyperplasia, and various vascular changes have been described [4–6]. Hepatic hemangioma is the most common benign hepatic lesion in the general population [7–9]. Its prevalence, radiological features, and clinical significance in SLE patients have not been sufficiently investigated. To date, only one study has specifically evaluated hepatic lesions in patients with SLE [10]. Furthermore, case reports and small series have described multiple or giant hemangiomas in SLE [8–15]. Although these case presentations and limited case-control studies exist, the true prevalence of hepatic hemangiomas in SLE cohorts remains unclear. Moreover, the association between hemangioma development and SLE disease activity, autoantibody profiles, and other clinical characteristics remains insufficiently clarified.

This study aimed to determine the frequency of hepatic hemangioma in patients with abdominal imaging in an SLE cohort, analyze the relationship between the presence of hemangioma and SLE disease characteristics such as disease duration and activity indices, and evaluate the radiological features and clinical outcomes of detected hemangiomas.

## Methods

### Study design and patient selection

This retrospective cohort study was conducted among SLE patients followed up at the Rheumatology Department of a tertiary university hospital from January 2000 to November 2025. The diagnosis of SLE was based on the 1997 American College of Rheumatology (ACR) revised criteria and/or the 2012 Systemic Lupus International Collaboration Clinics (SLICC) criteria [16, 17].

The whole SLE cohort (*n* = 402) was screened to identify patients who had undergone abdominal imaging (ultrasonography, contrast-enhanced/non-contrast computed tomography [CT], or magnetic resonance imaging [MRI]) for any reason. Of the 402 SLE patients followed at our center, only those who underwent abdominal imaging for clinical indications were included in the analysis. Therefore, the study population represents a selected subgroup rather than the entire SLE cohort.

Inclusion criteria were: ≥18 years of age, diagnosis of SLE, and availability of at least one abdominal imaging report. Exclusion criteria were: <18 years of age and patients who did not undergo abdominal imaging. Patient selection was not sequential and was based entirely on clinical indications (routine screening, abdominal pain, elevated liver enzymes, investigation of hematological abnormalities, etc.). Imaging indications were retrospectively recorded from patient files.

### Data collection and evaluation

Patients’ demographic data (age, gender), clinical characteristics (SLE diagnosis time, disease duration, follow-up duration, organ involvement [skin, renal, serositis, hematological involvement, etc.]), serological/laboratory findings (autoantibody profiles, complement [C] levels, etc.), and radiological reports were collected from the electronic patient record system. All patients underwent abdominal ultrasonography as the primary imaging modality. Contrast-enhanced CT and/or MRI was performed only when deemed necessary by the radiologist for further lesion characterization and to support differentiation between typical and atypical lesions. Radiological diagnoses were based on existing reports, and no centralized re-evaluation of imaging studies was performed. The diagnosis of hepatic hemangioma was made based on typical features in radiology reports (hyperechoic ultrasound, typical CT/MRI contrast dynamics: peripheral nodular contrast enhancement and central filling).

This retrospective observational study was approved by the Non-Interventional Clinical Research Ethics Committee of Eskişehir Osmangazi University Faculty of Medicine (Decision No: 2024-02, Date: 18.01.2024) and the study is reported in accordance with the Strengthening the Reporting of Observational Studies in Epidemiology (STROBE) statement [18].

### Statistical analysis

Continuous variables are expressed as mean ± standard deviation (for normally distributed data) or median (minimum–maximum), as appropriate. Categorical variables are presented as number (n) and percentage (%). Normality of continuous variables was assessed using the Shapiro–Wilk test. For intergroup comparisons, the independent samples t-test was used for normally distributed variables with equal variances, whereas the Mann–Whitney U test was applied for non-normally distributed continuous variables. Categorical variables were compared using the Pearson chi-square test or Fisher’s exact test, as appropriate. To identify associated factors of hepatic hemangioma, binary logistic regression analysis was performed. Variables with *p* < 0.20 in univariate analysis or considered clinically relevant were included in the multivariable analysis. Results are presented as odds ratios (OR) with 95% confidence intervals (CI). Model calibration was assessed using the Hosmer–Lemeshow goodness-of-fit test. No automated stepwise selection procedures were applied; instead, a clinically driven and parsimonious modeling approach was used. All statistical analyses were performed using IBM SPSS Statistics for Windows, Version 21.0 (IBM Corp., Armonk, NY, USA). A two-sided p value < 0.05 was considered statistically significant.

## Results

A total of 221 SLE patients who underwent abdominal imaging were included in the study. Tables 1, 2, 3, 4 and 5 summarizes demographic characteristics, clinical symptoms, laboratory results, and abdominal imaging data of the patients.

**Table 1:** Demographic characteristics, liver and spleen imaging features in SLE patients with and without hemangioma

Variables	Total	SLE patients with hemangioma +	SLE patients without hemangioma -	p-value
N	**221**	**26**	**195**	
Age, mean ± SD, years	49.62 ± 13.75 (n=221)	48.62 ± 8.52 (n=26)	49.75 ± 14.32 (n=195)	0.564
Age at SLE diagnosis, mean ± SD, years	36.29 ± 14.48 (n=221)	33.73 ± 10.86 (n=26)	36.64 ± 14.89 (n=195)	0.230
Gender, female, % (n/N)	86.4% (191/221)	80.8% (21/26)	87.2% (170/195)	0.554
Disease duration of SLE, mean ± SD, years	12.81 ± 8.14 (n=221)	15.85 ± 7.82 (n=26)	12.40 ± 8.12 (n=195)	**0.043**
Follow-up duration for SLE, mean ± SD, years	12.05 ± 7.97 (n=221)	15.38 ± 7.98 (n=26)	11.61 ± 7.89 (n=195)	**0.030**
SLEDAI score at SLE diagnosis, mean ± SD	8.24 ± 4.87 (n=219)	8.96 ± 4.66 (n=24)	8.15 ± 4.90 (n=195)	0.431
Timing of ultrasonography according to SLE disease duration	5.06 ± 5.09 (n=221)	7.20 ± 7.53 (n=26)	5.69 ± 4.47 (n=194)	**0.049**
Presence of hepatic hemangioma, % (n/N)	11.8% (26/221)	100% (26/26)	–	–
Timing of hemangioma detection relative to SLE diagnosis				
Concomitant, n, %	7, 27%	7, 27%	–	–
Before SLE diagnosis, n, %	1, 3.8%	1, 3.8%		
After SLE diagnosis, n, %	18, 69.2%	18, 69.2%		
Liver size, mean ± SD, cm	14.76 ± 2.28 (n=219)	14.72 ± 1.85 (n=26)	14.77 ± 2.33 (n=193)	0.903
Spleen size, mean ± SD, cm	10.82 ± 2.15 (n=217)	10.94 ± 2.28 (n=26)	10.80 ± 2.14 (n=191)	0.772
Hepatic steatosis, n (%)	35.2% (77/219)	26.9% (7/26)	36.3% (70/193)	0.473
Splenic infarction, n (%)	0.9% (2/217)	0.0% (0/26)	1.0% (2/191)	1.000

*SD* Standard deviation, *SLE* Systemic lupus erythematosus, *SLEDAI* Systemic lupus erythematosus disease activity index

Statistically significant values are shown bold in the tables

**Table 2:** Clinical features and laboratory findings at the time of SLE diagnosis in the total cohort and in patients with and without hemangioma

Variables	Total	SLE patients with hemangioma +	SLE patients without hemangioma -	p-value
N	**221**	**26**	**195**	
Cutaneous involvement, % (n/N)	56.1% (124/221)	57.7% (15/26)	55.9% (109/195)	1.000
Raynaud’s phenomenon, % (n/N)	12.2% (27/221)	11.5% (3/26)	12.3% (24/195)	1.000
Joint involvement, % (n/N)	51.6% (114/221)	46.2% (12/26)	52.3% (102/195)	0.703
Serositis, % (n/N)	10.4% (23/221)	19.2% (5/26)	9.2% (18/195)	0.220
Pericarditis, % (n/N)	5.4% (12/221)	3.8% (1/26)	5.6% (11/195)	1.000
Pleuritis, % (n/N)	7.2% (16/221)	19.2% (5/26)	5.6% (11/195)	**0.035**
Lung involvement, % (n/N)	1.4% (3/221)	0.0% (0/26)	1.5% (3/195)	1.000
Neurological involvement, % (n/N)	6.3% (14/221)	3.8% (1/26)	6.7% (13/195)	1.000
Hematological involvement, % (n/N)	49.3% (109/221)	57.7% (15/26)	48.2% (94/195)	0.484
Autoimmune hemolytic anemia, % (n/N)	20.9% (46/220)	38.5% (10/26)	18.6% (36/194)	**0.037**
Lymphopenia, % (n/N)	30.0% (66/220)	23.1% (6/26)	30.9% (60/194)	0.554
Leukopenia, % (n/N)	21.5% (47/219)	24.0% (6/25)	21.1% (41/194)	0.944
Thrombocytopenia, % (n/N)	21.8% (48/220)	19.2% (5/26)	22.2% (43/194)	0.930
Lupus nephritis, % (n/N)	21.1% (46/218)	30.8% (8/26)	19.8% (38/192)	0.302
Anemia, % (n/N)	51.4% (113/220)	42.3% (11/26)	49.5% (96/194)	0.632
Antiphospholipid syndrome, % (n/N)	12.7% (28/220)	15.4% (4/26)	12.4% (24/194)	0.753
Hemoglobin, mean ± SD, g/dL	11.85 ± 2.12 (n = 220)	11.38 ± 2.44 (n = 26)	11.92 ± 2.07 (n = 194)	0.288
Leukocyte count, mean ± SD, /10³/µL	5818.73 ± 2521.53 (n = 220)	5310.00 ± 1953.44 (n = 26)	5886.91 ± 2584.72 (n = 194)	0.183
Absolute lymphocyte count, mean ± SD, /10³/µL	1432.31 ± 831.06 (n = 170)	1346.00 ± 699.68 (n = 20)	1443.81 ± 848.42 (n = 150)	0.572
Absolute neutrophil count, mean ± SD, /10³/µL	3691.45 ± 1859.17 (n = 173)	3664.50 ± 1380.29 (n = 20)	3694.97 ± 1916.52 (n = 153)	0.930
Platelet count, mean ± SD, /10³/µL	210349.18± 106869.26 (n = 220)	206153.85 ± 82227.83 (n = 26)	210911.44± 109914.07 (n = 194)	0.346
Erythrocyte sedimentation rate, mean ± SD, mm/h	47.07 ± 35.15 (n = 202)	50.58 ± 41.44 (n = 26)	46.55 ± 34.23 (n = 176)	0.640
C-reactive protein, mean ± SD, mg/dL	4.50 ± 8.80 (n = 194)	4.79 ± 10.63 (n = 23)	4.46 ± 8.57 (n = 171)	0.887
Creatinine, mean ± SD, mg/dL	0.82 ± 0.38 (n = 214)	0.82 ± 0.35 (n = 24)	0.82 ± 0.38 (n = 190)	0.980
Aspartate aminotransferase, mean ± SD, U/L	28.46 ± 22.42 (n = 217)	22.64 ± 15.34 (n = 25)	29.21 ± 23.10 (n = 192)	0.067
Alanine aminotransferase, mean ± SD, U/L	24.66 ± 25.41 (n = 217)	18.96 ± 15.27 (n = 25)	25.40 ± 26.39 (n = 192)	0.080
ANA positivity, % (n/N)	100% (221/221)	100% (26/26)	100% (195/195)	1.00
Anti-ds-DNA positivity, % (n/N)	86.9% (192/221)	92.3% (24/26)	86.2% (168/195)	0.385
C3 hypocomplementemia, % (n/N)	58.4% (129/221)	80.8% (21/26)	55.4% (108/195)	**0.014**
C4 hypocomplementemia, % (n/N)	50.2% (111/221)	57.7% (15/26)	49.2% (96/195)	0.418
Anti-Sm positivity, % (n/N)	9% (20/221)	7.7% (2/26)	9.2% (18/195)	0.797
Anti-Sm/RNP positivity, % (n/N)	11.8% (26/221)	11.5% (3/26)	11.8% (23/195)	0.970
Anti-Ro positivity, % (n/N)	18.6% (41/221)	26.9% (7/26)	17.4% (34/195)	0.242
Anti-La positivity, % (n/N)	7.2% (20/221)	11.5% (3/26)	6.7% (13/195)	0.368

*ANA* Antinuclear antibody, *C* Complement, *SD* Standard deviation

Statistically significant values are shown bold in the tables

### Demographic characteristics, clinical symptoms, and laboratory features at the time of SLE diagnosis

The mean age of the study cohort was 49.62 ± 13.75 years, and 86.4% of the patients were female. The mean age at SLE diagnosis was 36.29 ± 14.48 years, and the mean disease duration was 12.81 ± 8.14 years (Table 1).

At SLE diagnosis, cutaneous involvement was observed in 56.1% of patients, hematological involvement in 49.3%, and lupus nephritis in 21.1%. Autoimmune hemolytic anemia (AIHA), pleuritis, and serositis were present in 20.9%, 7.2%, and 10.4% of patients, respectively. C3 hypocomplementemia was detected in 58.4%, while antinuclear antibody (ANA) and anti-dsDNA positivity rates were 100% and 86.9%, respectively. The mean hemoglobin level was 11.85 ± 2.12 g/dL, erythrocyte sedimentation rate (ESR) was 47.07 ± 35.15 mm/hour, and C-reactive protein (CRP) was 4.50 ± 8.80 mg/dL (Table 2).

**Table 3:** Distribution of indications for abdominal imaging (n=221 patients)

	n	%
Asymptomatic / routine screening / pre-renal biopsy evaluation	117	53.0
Abdominal pain	48	21.7
Cytopenia / hematological abnormalities / organomegaly	25	11.3
Elevated liver enzymes	18	8.2
Nausea-vomiting	5	2.2
Hematuria	2	0.9
Hypertension	2	0.9
Peripheral lymphadenopathy	2	0.9
Unknown cause	2	0.9

###  Frequency of hepatic hemangioma and indications for abdominal imaging

Hepatic hemangioma was detected in 11.8% (*n* = 26) of the 221 patients who underwent abdominal imaging. 69.2% of hemangiomas were detected after the diagnosis of SLE, 27% were concurrent, and only 3.8% were detected before the diagnosis of SLE. The most common indication for imaging was asymptomatic/routine screening/pre-renal biopsy evaluation (*n* = 117, 53%) (Table 3).

**Table 4:** Clinical and radiological characteristics of SLE patients with hepatic hemangioma (n = 26)

Characteristic	Value (n / % or mean ± SD)
Age (years)	Mean: 48.62 ± 8.52
	Median: 49
	Range: 35–63
Gender	Female: 21 (80.8%)
Imaging method	Ultrasonography 26 (100%)
	CT/MR additionally used in selected patients (n=3)
Liver size (cm)	Mean: 14.72 ± 1.85
	Median: 15.0
	Range: 11.2–16.2
Hemangioma characteristics	
Lesion type	Single: 17 (65.4%)
	Multiple: 9 (34.6%)
Morphology	Typical: 25 (96.2%)
	Atypical: 1 (3.8%)
Hemangioma size (cm)	Mean: 2.57 ± 2.22
	Median: 1.9
	Range: 0.4–11.2

*CT* Computed tomography, *MR* Magnetic resonance

### Radiological features of hepatic hemangioma

Among the 26 patients diagnosed with hepatic hemangioma, the mean age was 48.62 ± 8.52 years, and 80.8% were female. All hemangiomas were initially detected by abdominal ultrasound, with additional CT or MRI performed in some cases. The mean time from SLE diagnosis to hemangioma detection was 7.20 ± 7.53 years (median 4 years, range 0–20 years); for those diagnosed post-SLE (*n* = 18), the mean interval was 9.52 ± 6.56 years. Most hemangiomas were solitary (65.4%), and only one patient (3.8%) exhibited atypical features. The mean hemangioma size was 2.57 ± 2.22 cm, with a single giant lesion measuring 11.2 cm (Table 4).

### Comparison between patients with and without hemangioma

No significant differences were observed between patients with (*n* = 26) and without (*n* = 195) hepatic hemangioma regarding age, sex, age at SLE diagnosis, SLEDAI at diagnosis, liver and spleen size, hepatic steatosis, or splenic infarction (all *p* > 0.05). However, patients with hemangioma had significantly longer disease duration (15.85 ± 7.82 vs. 12.40 ± 8.12 years; *p* = 0.043) and follow-up duration (15.38 ± 7.98 vs. 11.61 ± 7.89 years; *p* = 0.030) compared with those without hemangioma (Table 1).

At SLE diagnosis, the prevalence of pleuritis was significantly higher in patients with hepatic hemangioma compared with those without (19.2% vs. 5.6%; *p* = 0.035). AIHA was also more frequent in the hemangioma group (38.5% vs. 18.6%; *p* = 0.037), and C3 hypocomplementemia was significantly more common (80.8% vs. 55.4%; *p* = 0.014). No significant differences were observed between the groups regarding other clinical manifestations, hematological parameters, inflammatory markers, liver enzymes, or autoantibody profiles (Table 2).

Comparison between patients with single (*n* = 17) and multiple (*n* = 9) hepatic hemangiomas revealed no significant difference in SLE disease duration. The mean disease duration was 16.00 ± 8.00 years (median 15.0, range 3–34) in the single hemangioma group and 15.56 ± 7.94 years (median 19.0, range 2–24) in the multiple hemangioma group (*p* > 0.05).

### Multivariate analysis/ associated factors of hepatic hemangioma

Binary logistic regression analysis was performed to identify associated factors with the presence of hepatic hemangioma. Variables that were statistically significant in univariate analysis or considered clinically relevant (age, gender, SLE disease duration, C3 hypocomplementemia, AIHA, and pleuritis) were included in the multivariable model. Due to significant multicollinearity with disease duration, C3 hypocomplementemia was not retained in the final model. Although three clinical variables (SLE disease duration, AIHA, and pleuritis) were initially entered into the multivariable model, only two variables remained statistically significant in the final model. In the final multivariable binary logistic regression model, SLE disease duration (OR = 1.06 per year; 95% CI: 1.01–1.12; *p* = 0.020), and AIHA at SLE diagnosis were independently associated with hepatic hemangioma (OR = 3.42; 95% CI: 1.32–8.88; *p* = 0.011). Pleuritis showed a borderline association (OR = 3.10; 95% CI: 0.93–10.30; *p* = 0.065), wheres age and gender were not independently associated (*p* > 0.05) (Table 5).

**Table 5:** Multivariable logistic regression analysis of factors associated with hepatic hemangioma

Variable	Coefficient (β)	Standard error	Odds ratio (OR)	95% confidence interval (CI)	p-value
Age	−0.01	0.02	0.99	0.95–1.02	0.512
Gender (Female)	0.29	0.61	1.33	0.40–4.40	0.641
SLE disease duration (years)	0.06	0.03	1.06	1.01–1.12	**0.020**
Presence of AIHA at SLE diagnosis	1.23	0.49	3.42	1.32–8.88	**0.011**
Presence of pleuritis at SLE diagnosis	1.13	0.61	3.10	0.93–10.30	0.065

Multivariable binary logistic regression model adjusted for age, gender, SLE disease duration, AIHA, and pleuritis

C3 hypocomplementemia was not retained in the final model due to multicollinearity with disease duration

*AIHA* Autoimmune hemolytic anemia, *SLE* Systemic lupus erythematosus

Statistically significant values are shown bold in the tables

## Discussion

The prevalence of hepatic hemangioma varies depending on the detection method in the general population. It has been reported at approximately 2–4% via ultrasound, up to 5% by CT, and 0.4–7.3% in autopsy series [8, 19, 20]. It is approximately five times more common in women than in men [8]. It peaks in the 30–50-year age range; most lesions are < 3–4 cm and asymptomatic [21]. Although liver involvement in SLE is most commonly associated with autoimmune hepatitis, drug-related toxicity, or vascular events, benign vascular lesions such as hepatic hemangioma and focal nodular hyperplasia have also increasingly reported in recent studies [1, 3, 10–13, 15, 22]. However, studies specifically evaluating hepatic hemangioma in SLE using radiological methods remain limited and are generally restricted to small cohorts or case-based reports. Our study represents one of the largest imaging-based SLE cohorts to date, based on the retrospective evaluation of abdominal imaging in 221 patients with SLE. However, given the absence of a non-SLE control group, this finding should not be interpreted as an increased prevalence or risk compared with the general population, and only reflects the frequency within an imaged SLE population. In a large Japanese autopsy registry including 1,468 SLE cases, hepatic hemangioma was reported in 1.5% of patients (23 cases) [23]. These lower rates may reflect methodological differences, including limited sensitivity of autopsy-based detection compared with modern imaging techniques. In a prospective ultrasound study of 35 SLE patients, benign hepatic lesions were reported in 54% of cases compared with 14% in healthy controls, and hepatic hemangioma was among the detected lesions. Although, the authors suggested a possible increased frequency of hemangioma in SLE (approximately fivefold), it should be noted that patients were not randomly selected; 65.7% of patients (*n* = 23) underwent routine screening/cyst follow-up, while the remainder underwent ultrasound due to the emergence of symptoms [10]. The results of this study should also be interpreted with caution, considering the potential for selection and detection biases. The indications for abdominal ultrasound in our study were predominantly asymptomatic/routine screening, follow-up, and pre-renal biopsy evaluation, followed by abdominal pain and cytopenia/organomegaly. This distribution is similar to the ultrasound indications in the study by Berzigotti et al. [10]. These results indicate a heterogeneous range of abdominal ultrasound indications in SLE, highlighting the need to discuss the cost-effectiveness of routine screening strategies. Our findings offer an imaging-based frequency estimate in one of the largest single-center SLE cohorts studied to date, situating it between lower rates reported in autopsy-based studies and higher rates observed in smaller prospective cohorts. The discrepancies across studies likely stem from differences in methodology, patient selection, and imaging approaches, rather than representing true epidemiological variation.

Hepatic hemangiomas are usually solitary, small (less than 5 cm), cavernous in shape, hyperechoic on ultrasound, and exhibit peripheral nodular enhancement with central progressive filling, the risk of symptoms or complications is minimal, and they typically don’t need to be monitored or treated. Single, multiple, cavernous, and giant hemangiomas have been described in SLE [11–14]. Berzigotti et al. identified 19 patients with hepatic hemangioma in their cohort of 35 SLE patients and reported a single lesion frequency of 63% in these patients [10]. In our series of 26 SLE patients with hepatic hemangioma, 65.4% (17/26) of the hemangiomas were solitary. This rate is almost completely consistent with the results of Berzigotti et al. Furthermore, one of our patients had a giant (11.2 cm) hepatic hemangioma, highlighting the size heterogeneity of SLE-associated hemangiomas.

To date, only one study has investigated risk factors for the development of hepatic hemangioma in SLE patients. In that prospective study, among 35 SLE patients with hepatic hemangioma, the presence of SLE was associated with an approximately fivefold increased risk for a single hemangioma and a fourfold increased risk for multiple hemangiomas, with multiple lesions being linked to longer SLE disease duration. The authors suggested that SLE may contribute to hemangioma development, noting that lesions were absent prior to SLE diagnosis in most patients (7 of 9), and proposed that chronic inflammation could trigger dysregulated angiogenesis [10]. Our study further demonstrated that SLE disease duration was significantly longer in patients with hepatic hemangioma. This finding is in line with previous reports suggesting an association between longer disease duration and multiple hemangiomas, supporting the hypothesis that cumulative vascular changes during the chronic course of SLE may contribute to hepatic hemangioma development. Chronic immune activation, sustained endothelial dysfunction, and progressive angiogenic remodeling may represent potential underlying mechanisms [10]. However, it is important to acknowledge that this association may also be influenced, at least in part, by detection bias. Patients with longer disease duration are more likely to undergo repeated medical evaluations and imaging studies over time, which may increase the probability of incidental hemangioma detection. In our cohort, although disease duration and follow-up duration were significantly longer in patients with hemangioma, the timing of ultrasound relative to SLE duration showed only a borderline difference, suggesting that detection bias may partially contribute but is unlikely to fully explain the observed association. In our subgroup analysis, disease duration did not differ significantly between patients with solitary and multiple hemangiomas. This may reflect the relatively long mean disease duration in our cohort (~ 16 years), patient heterogeneity, or differences in imaging indications. Notably, 69.2% of hemangiomas were detected after SLE diagnosis, with a small proportion identified prior to diagnosis, further suggesting that these lesions may emerge during the disease course, although the possibility of incidental pre-existing lesions cannot be excluded [10, 22].

Apart from SLE disease duration, the question of whether clinical and laboratory findings at the time of diagnosis can predict the presence of hepatic hemangioma has been only partially addressed in the literature. In a prospective study by Berzigotti et al., no significant association was observed between the presence or number of hemangiomas and parameters such as the SLEDAI score, specific autoantibodies, or overall disease activity; only acrocyanosis was more frequently detected in the hemangioma group [10]. In our study, pleuritis, AIHA, and C3 hypocomplementemia were more frequent at SLE diagnosis in patients with hepatic hemangioma. After multivariable adjustment, only AIHA remained independently associated, whereas pleuritis showed a non-significant trend and C3 hypocomplementemia lost significance. These findings suggest that hepatic hemangioma development in SLE may be associated not only to prolonged disease duration but also with specific early systemic immune manifestations. However, these associations should be interpreted with caution in terms of causality. The independent association with AIHA may reflect a more generalized and severe immune activation phenotype in SLE rather than a direct pathogenic role in vascular lesion formation. AIHA is typically associated with higher inflammatory burden, complement consumption, and systemic immune dysregulation, which may indirectly indicate an environment conducive to vascular alterations. Similarly, serosal inflammation such as pleuritis may represent systemic disease activity and immune activation, although it does not appear to be an independent associated factor of hepatic hemangioma in the adjusted model [10, 22]. Hormonal mechanisms should also be considered. Estrogen has been suggested as a trophic factor for hemangioma, and pregnancy or exogenous estrogen exposure may influence lesion growth [24]. Increased 16α-hydroxylation of estradiol has been demonstrated in SLE patients, and these metabolites may theoretically contribute to vascular proliferation beyond their uterotrophic effects [25, 26]. Future prospective studies incorporating detailed immunological and endocrinological evaluation may help clarify the pathophysiological pathways and improve risk stratification for hepatic vascular lesions in SLE.

The main strength of our study is that it represents, to our knowledge, the largest imaging-based cohort of SLE patients evaluating hepatic hemangioma frequency and associated factors, thereby contributing substantially to the existing literature. The relatively large sample size enhanced statistical power and enabled multivariable assessment of independent associated factors, particularly SLE disease duration and AIHA. Furthermore, detailed demographic, clinical, and laboratory comparisons were performed. These analyses suggest a potential association between specific early immunological manifestations and subsequent development of hepatic hemangioma, a relationship that has not been systematically explored in previous studies. However, our study also had several limitations. First, the retrospective design inherently limits causal inference and may introduce information bias. Second, selection bias cannot be excluded, as abdominal imaging was performed based on clinical indications, most commonly routine screening or abdominal symptoms. Therefore, the reported frequency reflects a selected population and does not represent the true prevalence in the overall SLE cohort. In addition, patients with longer disease duration may have undergone more frequent abdominal imaging over time, potentially increasing the likelihood of incidental hemangioma detection. Therefore, the observed association between disease duration and hemangioma presence may partly reflect surveillance bias rather than a direct biological relationship. Histopathological confirmation was not available, and diagnoses were based on characteristic radiological findings. Furthermore, the absence of a matched non-SLE control group precludes any direct comparison with the general population and limits conclusions regarding relative risk. Accordingly, comparisons with published literature were used solely for contextual interpretation and should not be interpreted as evidence of increased prevalence or risk. Another limitation is the lack of standardized longitudinal imaging, precluding assessment of incident hemangioma development over time. Finally, as a single-center study, the generalizability of our findings may be limited.

## Conclusions

In conclusion, hepatic hemangiomas may be incidentally detected in a subgroup of patients with SLE undergoing abdominal imaging, particularly in those with longer disease duration and a history of AIHA at SLE diagnosis. However, these findings should be interpreted with caution due to the retrospective design, potential selection and detection biases, the absence of systematic imaging in the overall SLE cohort, and the lack of a control group. Prospective, multicenter studies with standardized, longitudinal, and controlled imaging protocols, along with detailed immunological assessment, are needed to further clarify the true prevalence and clinical significance of hepatic hemangioma-like vascular lesions in SLE.

## Supplementary Information

Below is the link to the electronic supplementary material.

Supplementary Material 1

## Data Availability

Not applicable.
