# Supplementary material for: Frequency and associated factors of hepatic hemangioma in systemic lupus erythematosus patients undergoing abdominal imaging: a retrospective cohort study
Source: Rheumatol Int. 2026 May 28;46(6):119. doi: 10.1007/s00296-026-06164-7 (PMC13219105; doi:10.1007/s00296-026-06164-7)
Supplement: Supplementary file 1 — Supplementary Material 1 [file 296_2026_6164_MOESM1_ESM.docx]

Title: **Frequency and Associated Factors of Hepatic Hemangioma in Systemic Lupus Erythematosus Patients Undergoing Abdominal Imaging: A Retrospective Cohort Study**

Supplementary Material A

STROBE Checklist for Reporting Observational Studies (Cohort Design)

| **Item No** | **Recommendation** | **Reported on Section** | **Page No.** | **Status** |
| --- | --- | --- | --- | --- |
| **Title and Abstract** |  |  |  |  |
| 1a | Indicate the study’s design with a commonly used term in the title or the abstract | Title | 1 | Yes |
| 1b | Provide in the abstract an informative and balanced summary of what was done and what was found | Abstract | 1 | Yes |
| **Introduction** |  |  |  |  |
| 2 | Explain the scientific background and rationale for the investigation | Introduction | 2 | Yes |
| 3 | State specific objectives, including any prespecified hypotheses | Introduction (last paragraph) | 2 | Yes |
| **Methods** |  |  |  |  |
| 4 | Present key elements of study design early in the paper | Methods (Study Design and Patient Selection) | 3 | Yes |
| 5 | Describe the setting, locations, and relevant dates, including periods of recruitment, exposure, follow-up, and data collection | Methods (Study Design and Patient Selection) | 3 | Yes |
| 6a | Give the eligibility criteria, and the sources and methods of selection of participants | Methods (Study Design and Patient Selection) | 3,4 | Yes |
| 7 | Clearly define all outcomes, exposures, predictors, potential confounders, and effect modifiers | Methods (Data Collection and Evaluation) | 3,4 | Yes |
| 8 | For each variable of interest, give sources of data and details of methods of assessment | Methods (Data Collection and Evaluation) | 3, 4 | Yes |
| 9 | Describe comparability of assessment methods if there is more than one group | N/A | - | N/A |
| 10 | Describe any efforts to address potential sources of bias | Methods + Discussion | 7-10 | Yes |
| 11 | Explain how the study size was arrived at | Methods | 3 | Yes |
| 12a | Describe all statistical methods, including those used to control for confounding | Statistical Analysis | 4 | Yes |
| 12b | Describe any methods used to examine subgroups and interactions | Statistical Analysis + Results | 4 | Yes |
| 12c | Explain how missing data were addressed | Statistical Analysis | - | Not reported |
| 12e | Describe any sensitivity analyses | — | - | Not reported |
| 13a | Report numbers of individuals at each stage of the study | Results (first paragraph) | 4-6 | Yes |
| 13b | Give reasons for non-participation at each stage | Methods | 3,4 | Yes |
| 13c | Consider use of a flow diagram | — | - | Not provided |
| **Results** |  |  |  |  |
| 14a | Give characteristics of study participants | Results + Table 1 | 4,5,6,Tables | Yes |
| 14b | Indicate number of participants with missing data for each variable | Table 1, Table 2 | Tables | Yes |
| 14c | Summarise follow-up time | Results | 4-6 | Yes |
| 15 | Report numbers of outcome events or summary measures | Results | 4-6 | Yes |
| 16a | Give unadjusted estimates and, if applicable, confounder-adjusted estimates and their precision | Results + Table 5 | 4-6, Tables | Yes |
| 17 | Report other analyses performed (e.g., subgroup analyses) | Results | 4-6 | Yes |
| **Discussion** |  |  |  |  |
| 18 | Summarise key results with reference to study objectives | Discussion | 7-10 | Yes |
| 19 | Discuss limitations of the study, taking into account sources of potential bias | Discussion | 10 | Yes |
| 20 | Give a cautious overall interpretation of results | Discussion + Conclusions | 11 | Yes |
| 21 | Discuss the generalisability of the study results | Discussion + Conclusions | 7-10 | Yes |
| **Other information** |  |  |  |  |
| 22 | Give the source of funding and the role of the funders | — | 11 | This study received no funding. The article processing charge (Open Access fee) was supported by TÜBİTAK. |
